# Genome- and Transcriptome-Wide Identification of C3Hs in Common Bean (*Phaseolus vulgaris* L.) and Structural and Expression-Based Analyses of Their Functions During the Sprout Stage Under Salt-Stress Conditions

**DOI:** 10.3389/fgene.2020.564607

**Published:** 2020-09-15

**Authors:** Qi Zhang, Wen-jing Zhang, Zhen-gong Yin, Wei-jia Li, Hao-hao Zhao, Shuo Zhang, Lin Zhuang, Yu-xin Wang, Wen-Hui Zhang, Ji-Dao Du

**Affiliations:** ^1^Laboratory Crop Genetics and Breeding, College of Agriculture, Heilongjiang Bayi Agricultural University, Daqing, China; ^2^Crop Resources Institute of Heilongjiang Academy of Agricultural Sciences, Heilongjiang, China; ^3^Laboratory Crop Genetics and Breeding, National Coarse Cereals Engineering Research Center, Daqing, China

**Keywords:** *C3H* gene family, common bean, sprout stage, salt stress, gene expression analysis, transcriptome

## Abstract

CCCH (C3H) zinc-finger proteins are involved in plant biotic and abiotic stress responses, growth and development, and disease resistance. However, studies on *C3H* genes in *Phaseolus vulgaris* L. (common bean) are limited. Here, 29 protein-encoding *C3H* genes, located on 11 different chromosomes, were identified in *P. vulgaris*. A phylogenetic analysis categorized the *PvC3H*s into seven subfamilies on the basis of distinct features, such as exon–intron structure, *cis*-regulatory elements, and MEME motifs. A collinearity analysis revealed connections among the *PvC3H*s in the same and different species. The *PvC3H* genes showed tissue-specific expression patterns during the sprout stage, as assessed by real-time quantitative PCR (RT-qPCR). Using RNA-sequencing and RT-qPCR data, *PvC3H*s were identified as being enriched through Gene Ontology and Kyoto Encyclopedia of Genes and Genomes analyses in binding, channel activity, and the spliceosome pathway. These results provide useful information and a rich resource that can be exploited to functionally characterize and understand *PvC3H*s. These *PvC3Hs*, especially those enriched in binding, channel activity, and the spliceosome pathway will further facilitate the molecular breeding of common bean and provide insights into the correlations between *PvC3Hs* and salt-stress responses during the sprout stage.

## Introduction

The zinc transcription factor family is among the largest in eukaryotes (Li et al., 2001). Zinc finger proteins have various functions, such as DNA and RNA binding and transcriptional activation, as well as being involved in protein–protein interactions (Gao et al., 2002). The zinc-finger motif is composed of one or more cysteine residues and one histidine residue. This superfamily is divided into nine categories on the basis of the numbers and distances between conserved cysteine and histidine residues (Wolfe et al., 2000). However, there are limited studies on CCCH (C3H) zinc-finger proteins. C3H type zinc-finger proteins typically possess one or multiple zinc-finger motifs, characterized by three cysteine residues followed by a histidine residue, and they account for approximately 0.8% of all zinc-finger proteins (Hall, 2005).

In plants, at present, the functions of only some C3H zinc-finger proteins have been revealed. The *C3H* gene families are involved in various plant developmental and adaptation-related processes and play important roles in hormone regulation. In the jasmonic acid (JA) pathway of rice (*Oryza sativa*), the overexpression of *OsDOS* results in negative regulation that significantly delays leaf senescence (Kong et al., 2006). In *Arabidopsis thaliana*, *ATML1* is a specific *C3H* gene that is indispensable for the embryogenesis of seeds (Li, 1998). *AtC3H14* and *AtC3H15* regulate secondary wall thickening, anther development, and male fertility (Kim et al., 2014; Lu et al., 2014; Chai et al., 2015). Additionally, some C3H zinc-finger proteins participate in abiotic and biotic stress responses. In *A. thaliana*, *TZF1* is involved in sugar signal transduction, and it’s overexpression delayed an increase in salt tolerance (Lin et al., 2014). *AtZFP1* improves plant salt tolerance (Han et al., 2014) by maintaining the ion balance and alleviating oxidation and osmotic stress. *AtSZF1* and *AtSZF2* modulate the expression of many salt-sensitive genes, resulting in enhanced salt tolerance in *A. thaliana* (Sun et al., 2007). Rice *C3H12* regulates rice bacterial blight resistance (Deng et al., 2012) through the JA signaling pathway, and after the over-expression of *C3H18* in sweet potato, plant resistance to drought and high salt conditions is enhanced (Zhang et al., 2019) through the regulation of many stress-response genes. In cotton (*Gossypium hirsutum*), *ZFP1* controls salt tolerance, as well as disease resistance, by interacting with dehydrin and disease resistance-related proteins, respectively (Guo et al., 2009).

*Phaseolus vulgaris* L. is a valuable legume crop and a livelihood source for many people (Broughton et al., 2003; Wu et al., 2020). Additionally, the crop is rich in proteins and micro-nutrients that are essential for human health. The crop yield has slowly improved, particularly in tropical regions, and favorable yields have been realized in temperate countries because of the development of new cultivars (Rosenzweig and Liverman, 1992). The *C3H* genes participate in many responses related to various abiotic stresses in several plant species. However, the *C3H* family in *P. vulgaris* has not been characterized. To screen for potential salt-tolerance genes expressed during the sprout stage of *P. vulgaris*, members of *C3H* gene family were identified at the genome level. Here, 29 *PvC3H* members were detected in the *P. vulgaris* genome. Furthermore, their distribution among the chromosomes was determined and their conserved domains and collinearity investigated. *PvC3H* gene expression levels were evaluated in multiple *P. vulgaris* varieties. Additionally, the expression patterns of the *PvC3H* genes were analyzed in various tissues at the sprout stage, and the possible regulation of specifically expressed *PvC3H* genes under salt stress and other stress conditions was investigated. These findings offer crucial information for studying the evolution and functional differentiation of the *C3H* gene family in common bean.

## Materials and Methods

### Identification and Phylogenetic Analysis of C3H Genes in *P. vulgaris*

The HMMER software^1^ and the Pfam protein family database^2^ (Finn et al., 2016) were employed to identify candidate C3H proteins containing the C3H domain (PF00642). The protein annotation file was retrieved from the website of Esembl plants^3^. Subsequently, InterPro^4^ (Finn et al., 2017) and SMART^5^ (Letunic et al., 2015; Han et al., 2019) software were applied to verify the reliability of the C3H domain prediction. Lastly, Interpro^6^, WoLF PSORT^7^, P3DB^8^, and the ExPASy Proteomics Server^9^ were employed to verify the integrity of the C3H domains in the candidate genes. Each *C3H* gene was named on the basis of their precise position on the chromosome.

The C3H protein sequences of the other species, like *Arabidopsis*, were obtained from Esembl plants using the C3H domain. C3Hs were introduced into MEGA X to perform multiple sequence alignments. The Maximum-likelihood method with the JTT + G model, which MEGA X predicted as the optimal model, and 1,000 replicates were used to produce bootstrap values. MEGA X was used to revise and construct a phylogenetic tree.

### Analyses of Gene Structure and Conserved Motifs, and Promoter Predictions

The GSDS platform^10^ (Guo et al., 2007) was used to display and analyze the exon–intron structures of the *PvC3H* genes. GeneWise (Birney et al., 2004) was used to examine the correspondence to coordinates in the DNA (comprising both exons and introns) versus protein sequences. Subsequently, in-house perl scripts were used to transform the C3H domain coordinates in the protein sequence to those in the nucleotide sequence. The intron-splicing phase in the hinge and the basic regions of the C3H domains from all the *PvC3H* genes were characterized and separated into various types. The MEME tool^11^ (Bailey et al., 2009) was employed to detect additional motifs outside the C3H domain in the protein sequences. Motifs containing 10–50 amino acids and *E*-value < 1e^–20^ were characterized. All of the motifs found among the PvC3Hs were compared to identify the group-specific or group-conserved signatures. The motifs were numbered based on the order in which they occurred in the protein sequences.

### Collinearity Analysis of *PvC3Hs*

The mapping of the *PvC3H*s to the chromosomes was performed using the chromosomal locations provided by Esembl plants. The Multiple Collinearity Scan toolkit (MCScanX) was used to examine gene duplication events based on default parameters (Wang et al., 2012). Additionally, circos (version 0.69^12^) was used to generate the visualization as described previously (Krzywinski et al., 2009).

### Salt-Tolerant and Salt-Sensitive Common Beans Exposed to Different Treatments

Two kinds of common beans, which are salt-tolerant (R) and salt-sensitive (N), respectively, were selected as the test materials. They were treated with water (W) and 0.4% NaCl (S). The plants were then placed in a 28°C incubator without light for 7 days. The numbers of germination events were recorded consecutively for 7 days.

### Transcriptome Analysis

After 5 days, the bean-free hypocotyls of WN, SN, WR, and SR were selected, with three repeated treatments. All the materials were rapidly frozen in liquid nitrogen at a low temperature and then rapidly ground independently into powder. The RNAs were extracted using TRIzol (Invitrogen, Carlsbad, CA, United States), and the RNA quality was determined using 1% agarose gel electrophoresis and NanoDrop (Thermo, Carlsbad, CA, United States) instrumentation. The concentration and purity of the RNAs were determined using a NanoDrop spectrophotometer. The OD_260__/__280_ was required to be between 1.8 and 2.2, and that of the 28S/18S was required to be 1.0 or greater. An Agilent Bioanalyzer 2100 (Agilent Technologies, Carlsbad, California) was used to determine the RNA integrity. All the sequencing samples were rated as A-level, meeting the requirements of library construction. After qualified RNA detection, sequencing libraries were constructed (Supplementary Table 1) on an Illumina HiSeq 2500 PE 150 and referring to the NCBI Common bean (P. vulgaris) reference genome. The heat map was constructed using Tbtools software (Chen et al., 2020). The Kyoto Encyclopedia of Genes and Genomes (KEGG) database^13^ and the phytozome database^14^ were used for gene annotation. Gene ontology (GO) annotations were predicted using Blast2GO software (Conesa et al., 2005), and the functional classification of genes was performed using WEGO software (Ye et al., 2006).

### Quantitative Real-Time PCR (RT-qPCR) Analysis of *PvC3Hs*

Different tissues (cotyledon, hypocotyl, and radicle) of WR were selected on the fifth day during the sprout stage, and hypocotyls of the four samples (WR, WN, SR, and SN) were selected on the fourth, fifth and sixth days. Each sample had three biological repeats. Total RNA was extracted using RNA isolater Total RNA Extraction Reagent (Vazyme Biotech, China). After the extraction, the RNA samples were electrophoresed in 1% agarose gel for detection and quality control. Additionally, a NanoDrop spectrophotometer was used to check the concentration and purity of the RNAs. Samples of poor quality were discarded. Premier 5.0 (Pi et al., 2018) was used to design the RT-qPCR primers (Supplementary Table 2). *PvC3H*s were selected from the GO enrichment and KEGG pathways, and some *PvC3H*s were selected as candidate genes for verification. *ACTIN-11* of common bean served as an internal control gene (Borges et al., 2012). The RNA was transcribed to cDNA, and then, RT-qPCR was performed using 2 × ChamQ Universal SYBR qPCR Master Mix Kit (Vazyme Biotech, China). The RT-qPCR was performed in triplicate. The relative gene expression was determined as follows (Livak and Schmittgen, 2001):

Relative **E**xpression = 2^Δ^ ^Δ^ ^Ct^, **W**here ^Δ^ ^Δ^Ct = [Ct(*Pv* target genes) −Ct(*PvACTIN-11*)].

### Data Analyses

Office 2013 and SPSS 19.0 were used for data analyses (Li et al., 2018).

## Results

### Identification of C3H Family Members in *P. vulgaris* L.

In total, 65 protein sequences with C3H domains were identified using a HMM profile analysis from the common bean genome. Furthermore, InterPro and SMART analyses assigned 29 putative members, PvC3H01–29, to the common bean C3H family (Table 1). The 29 PvC3H members were located on the 11 chromosomes (Chrs) of common bean.

**TABLE 1:** Information regarding *Phaseolus vulgaris* L. C3H family members.

Gene name	Gene Bank	Gene ID	chr	Location	Protein length	CDS length	Lsoelectric point	Molecular weigth	Instability index	Aliphatic index
*PvC3H01*	ESW34310.1	*Phvul.001G141900*	1	39106940:39110116	295	888	9.31	31402.61	38.53	57.66
*PvC3H02*	ESW34844.1	*Phvul.001G186200*	1	45111242:45114277	315	948	9.74	36528.44	71.26	47.05
*PvC3H03*	ESW28984.1	*Phvul.002G034300*	2	3447337:3452995	699	2100	6.19	77929.46	49.69	72.42
*PvC3H04*	ESW25121.1	*Phvul.003G009100*	3	904447:908755	258	777	9.79	30270.78	81.33	53.33
*PvC3H05*	ESW26238.1	*Phvul.003G102600*	3	25189363:25194111	483	1452	8.62	50645.69	65.52	54.53
*PvC3H06*	ESW26651.1	*Phvul.003G136700*	3	32863086:32868451	601	1806	6.30	66954.69	47.72	72.38
*PvC3H07*	ESW26973.1	*Phvul.003G162600*	3	37078874:37081522	344	1035	8.26	38457.22	47.77	55.32
*PvC3H08*	ESW27506.1	*Phvul.003G207900*	3	42243339:42248077	485	1458	4.89	52877.12	58.05	48.02
*PvC3H09*	ESW28409.1	*Phvul.003G284200*	3	51035876:51040126	376	1131	6.56	39672.21	50.24	42.85
*PvC3H10*	ESW24345.1	*Phvul.004G122600*	4	39407374:39416501	424	1275	7.86	46083.01	22.55	75.57
*PvC3H11*	ESW20849.1	*Phvul.005G019900*	5	1724921:1727678	363	873	6.94	40635.13	55.13	70.69
*PvC3H12*	ESW22104.1	*Phvul.005G127700*	5	35217300:35220277	308	1146	9.24	36262.07	81.14	48.47
*PvC3H13*	ESW22107.1	*Phvul.005G127800*	5	35227175:35228656	290	873	9.06	34119.87	76.75	53.10
*PvC3H14*	ESW18515.1	*Phvul.006G047900*	6	16181121:16187473	496	1491	5.31	55805.75	51.79	74.15
*PvC3H15*	ESW19882.1	*Phvul.006G163300*	6	27430746:27431063	483	1452	8.49	50620.70	64.74	55.55
*PvC3H16*	ESW20584.1	*Phvul.006G221300*	6	31916147:31924712	565	1698	6.40	66815.55	56.23	44.69
*PvC3H17*	ESW14610.1	*Phvul.007G002700*	7	186479:191473	298	897	9.34	31581.67	38.29	56.71
*PvC3H18*	ESW14969.1	*Phvul.007G033200*	7	2618882:2622003	365	1098	8.00	40310.00	49.76	62.77
*PvC3H19*	ESW15970.1	*Phvul.007G118500*	7	19087659:19092941	444	1335	8.86	48721.00	55.66	59.37
*PvC3H20*	ESW16542.1	*Phvul.007G165200*	7	39536306:39540749	466	1401	8.97	49805.21	57.55	64.23
*PvC3H21*	ESW17023.1	*Phvul.007G203900*	7	44306014:44306584	125	378	8.63	14241.05	53.39	53.84
*PvC3H22*	ESW17675.1	*Phvul.007G259300*	7	49744488:49748916	293	882	9.37	30682.19	43.58	64.37
*PvC3H23*	ESW11726.1	*Phvul.008G054800*	8	4888914:4897088	426	1281	8.41	46803.62	65.21	53.40
*PvC3H24*	ESW13654.1	*Phvul.008G214600*	8	52694068:52694121	427	1284	7.86	46764.29	57.51	53.51
*PvC3H25*	ESW08797.1	*Phvul.009G075500*	9	12478833:12479986	251	756	8.30	27883.32	64.45	59.12
*PvC3H26*	ESW09958.1	*Phvul.009G169600*	9	24777928:24782632	492	1479	4.81	53385.37	58.93	50.30
*PvC3H27*	ESW06876.1	*Phvul.010G084000*	10	31414254:31417666	329	990	6.58	37373.60	49.50	50.21
*PvC3H28*	ESW03434.1	*Phvul.011G013700*	11	1051759:1053851	700	2103	5.96	76237.53	54.75	68.60
*PvC3H29*	ESW05458.1	*Phvul.011G180700*	11	45672467:45688469	484	1455	7.51	52859.50	54.37	56.92

**FIGURE 1:**
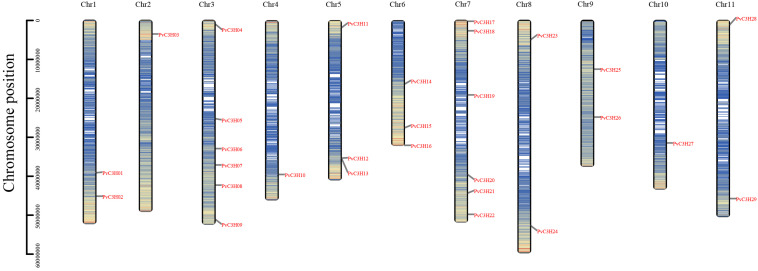
Chromosomal localization of *PvC3H* members. The *y*-axis indicates chromosome position. The denser the blue, the greater the gene density of the area.

Specifically, most of the *PvC3H*s were located on Chrs 7 and 3, while few genes were distributed on Chrs 2 and 10 (Figure 1). At least one PvC3H member was mapped to each chromosome.

### Phylogenetic Analysis of C3H Proteins

**FIGURE 2:**
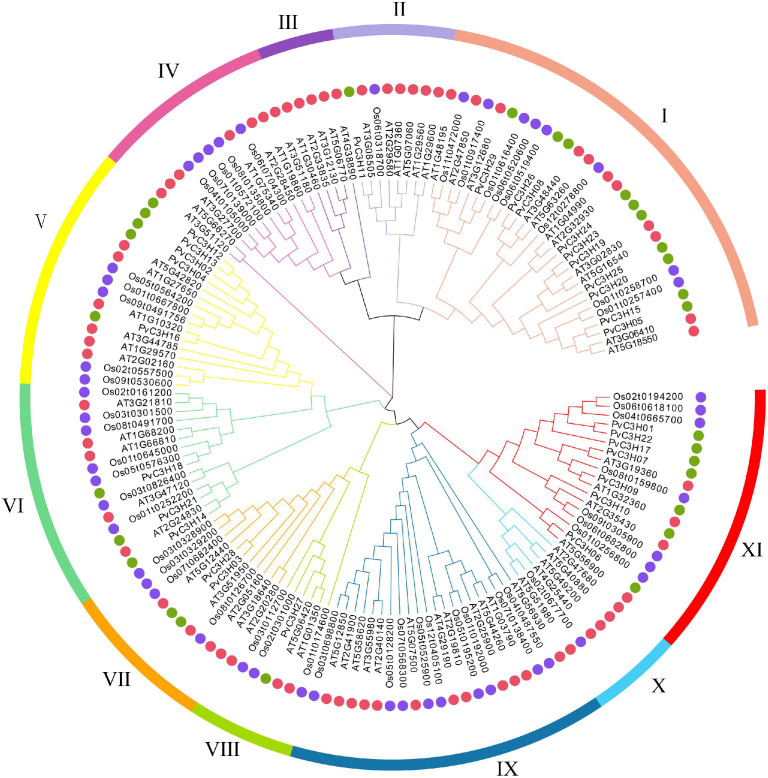
Phylogenetic tree showing the distribution of C3H proteins from three plant species: *P. vulgaris* L., *O. sativa*, and *A. thaliana*. The ML method with the JTT + G model was used to construct the phylogenetic tree in MEGA X. The proteins were divided into 11 subfamilies (I–X I) delineated by different colors. The red, purple, and green circles represent the *A. thaliana*, *O. sativa*, and *P. vulgaris* L. C3H family members, respectively.

A comprehensive phylogenetic tree comprising 147 C3H protein sequences from three plants, 29 sequences from common bean (*P. vulgaris*), 50 from rice (*O. sativa.*), and 68 from the *A. thaliana*, was constructed (Figure 2). The PvC3H, rice protein sequences and *A. thaliana* C3H sequences were obtained from a public database retrieved using the pfam method. The phylogenetic tree of the C3H sequences was generated with the Maximum-likelihood method using a JTT + G model, which was the optimal model as determined by MEGA X software. Using the phylogenetic tree and the classification method applied to the *C3H* genes in *A. thaliana* (Wang et al., 2008), which were separated into 11 subfamilies, I–X I, the *C3H* genes in *P.* were classified into only seven subfamilies (I, III, V, VI, VII, VIII, and XI).

### Gene Structures and Conserved Motif Compositions of the *PvC3Hs*

**FIGURE 3:**
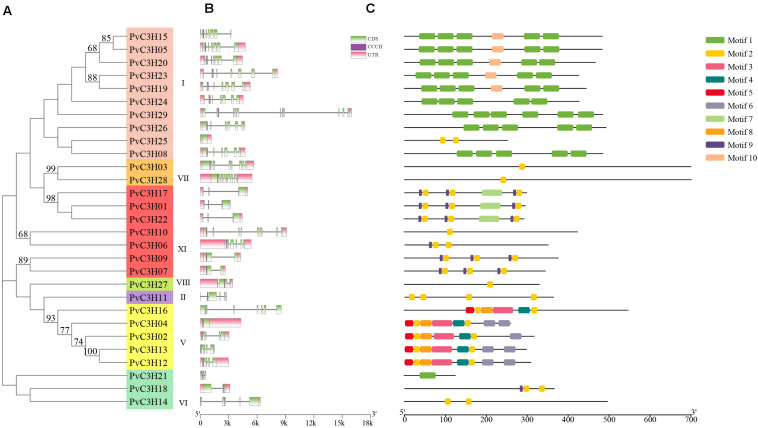
Gene structures and motifs of *PvC3H* family members. **(A)** The clustering of PvC3H proteins based on a Maximum-likelihood based phylogenetic tree. Bootstrap values from 1,000 replicates are shown at each node. **(B)** *PvC3H* structures. The purple boxes represent C3H domains, the green boxes represent the UTR regions, the yellow boxes represent the exons, and the black lines represent the introns. **(C)** Schematic depiction of 10 conserved motifs in PvC3H proteins. The MEME online tool was used to identify motifs in the PvC3H proteins. Each motif type is denoted using different-colored blocks, and the numbers in the boxes (1–10) signify motifs 1–10. The length and position of each colored box is scaled to size.

The exon–intron structures of the 29 *PvC3Hs* were examined to reveal the gene structures and the evolutionary trajectory (Figure 3A). Each subfamily had a conserved C3H domain, and the exon and intron numbers, ranging from 0 to 11, were similar in each subfamily. Generally, the *PvC3H*s that clustered together showed similar structures (Figure 3B).

The conservative motifs of the 29 *PvC3H*s were analyzed using MEME software. Figure 3C shows the motifs and the composition of each *PvC3H*. The motifs of each subfamily were relatively similar, which confirmed the close associations among the same subfamilies in the evolutionary tree.

### *Cis*-Regulatory Element Analysis of *PvC3Hs*

**FIGURE 4:**
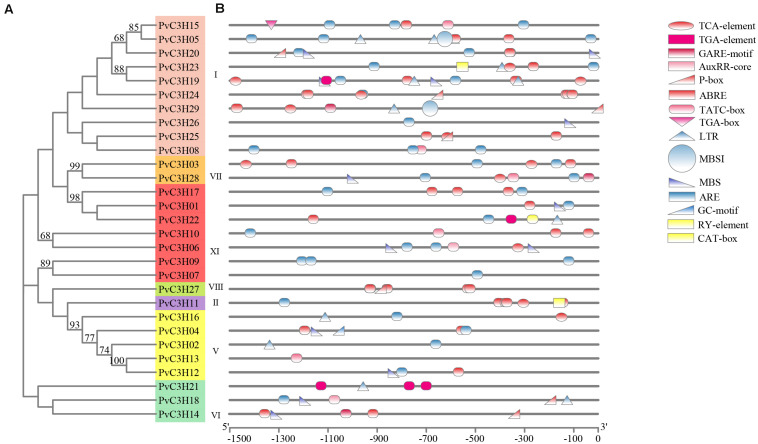
*Cis*-regulatory element analysis of the *PvC3H* family. **(A)** The clustering of PvC3H proteins based on a ML phylogenetic tree. Bootstrap values from 1,000 replicates are shown at each node. **(B)** *Cis-*element analysis of the promoter regions of *P. vulgaris C3H* genes. Different colored shapes represent different kinds of *cis*-regulatory elements. Red, blue, and yellow represent hormone-, stress-, and seed germination-related elements, respectively.

The *cis*-regulatory elements were analyzed using PlantCARE software, which showed the promoters of *PvC3H*s as determined by comparison with the common bean reference genome database. In total, 13 elements, including hormone-forming, stress-related, and seed germination elements, were found, as shown in Figures 4A,B and Supplementary Table 3. The phytohormone responsive cis-acting elements (red) may be activated during plant growth, which suggests that the *PvC3H* family responds to stress-related hormones. Some of the elements present are induced by low-temperature and drought, indicating that the *PvC3H*s may have roles in abiotic stress responses. Other elements (yellow) are involved in plant germination or activated during the sprout stage, which indicates that these elements play roles during the plant germination period.

### Collinearity Analysis and the Tandem Replication of *PvC3Hs*

**FIGURE 5:**
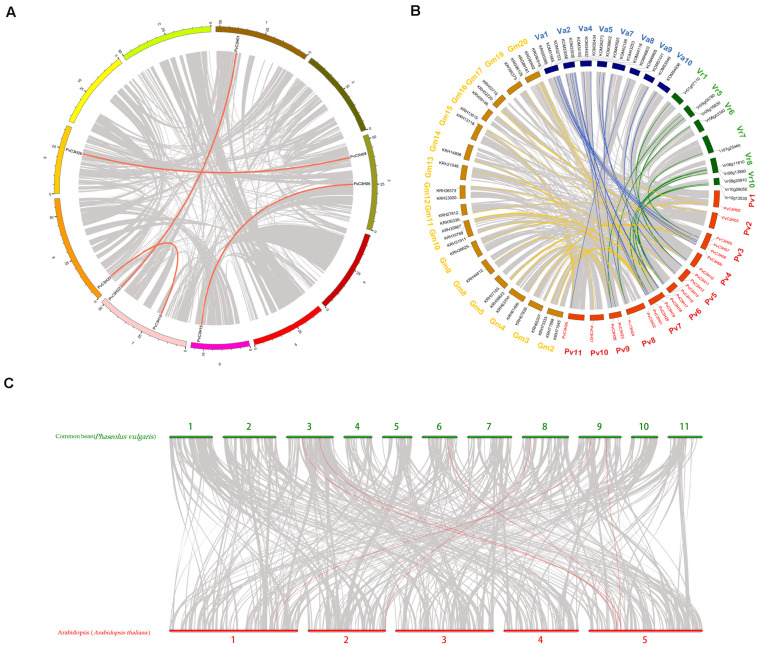
Collinearity analysis of the *C3H* gene family in *P. vulgaris*. The bright lines represent collinearity, and the gray lines represent all isomorphic blocks in the genome. The collinear relationship **(A)** within the common bean species, **(B)** between common bean and three other species of legumes, and **(C)** between common bean and *A. thaliana*.

The collinear analysis of the *P. vulgaris C3H* family of genes within species and with other legume crops and *A. thaliana* are shown in Figure 5. Only four pairs of *C3H* family genes in *P. vulgaris* were collinear. In leguminous species, there was collinearity between the *C3H* family genes and 43 genes in soybean (*Glycine max*), 23 genes in adzuki bean (*Vigna angularis*), and 11 genes in mung bean (*Vigna radiata*), which indicated that the *P. vulgaris C3H* family genes were closely related to genes in the legume family. Additionally, *PvC3H05* and *PvC3H26* were collinear with *AT5G18550*, and *PvC3H08* and *PvC3H15* were collinear with *AT5G16540*, indicating that the four *C3H* family genes may have similar functions in *P. vulgaris*. Selection pressure analyses of collinear genes have revealed that a ratio of the non-synonymous mutation rate (Ka) to the synonymous mutation rate (Ks) = 1 usually represents neutral selection, whereas Ka/Ks < 1 represents negative or purified selection. Supplementary Table 4 shows that the Ka/Ks values of most pairs of replicated *CH3* genes in *P. vulgaris* were less than 1, indicating that they underwent purification and selection after replication, and we speculated that their gene functions did not undergo differentiation, thereby largely maintaining the functional similarities of the *PvC3H* family members.

### Variation in the Germination of R and N Common Beans

**FIGURE 6:**
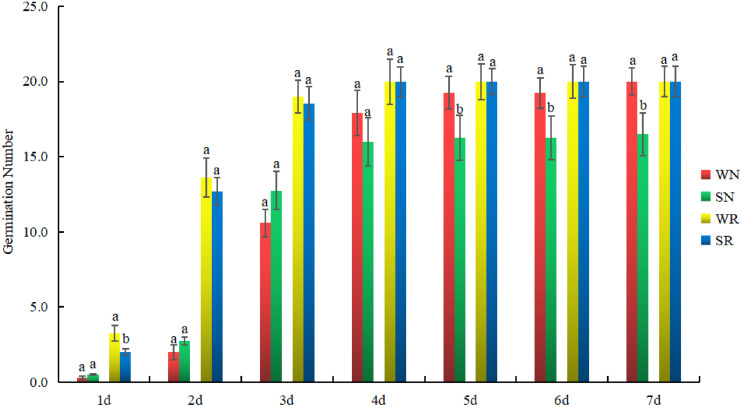
Dynamic variation in germination numbers of salt-tolerant (R) and salt-sensitive (N) common bean varieties. They were treated with water (W) and 0.4% NaCl (S). Green and red bars represent WN and SN, respectively, while yellow and blue bars represent WR and SR, respectively. The *x*-axis indicates time, and the *y*-axis indicates the number of germination events. Lower case letter(s) above bars indicate significant differences (α = 0.05, LSD) among the treatments. The *x*-axis indicates germination time, and the *y*-axis indicates germination number.

To clarify the sampling time, the germination numbers of N and R were investigated. The number of germination events for R after each treatment was similar. There was no difference in the number of germination events under S conditions. For N, a significant difference in the number of germination events was found between the two treatments. The difference appeared on the fifth day. The number of germination events between WN and SN differed after 5 day. There was no difference in the germination number of R after 5 days of the W treatment compared with the S treatment, but the germination number of N had decreased (Figure 6). Therefore, the fifth day was used as the sampling time.

### Expression Analysis of PvC3Hs in Different Tissues of Common Bean During the Sprout Stage

**FIGURE 7:**
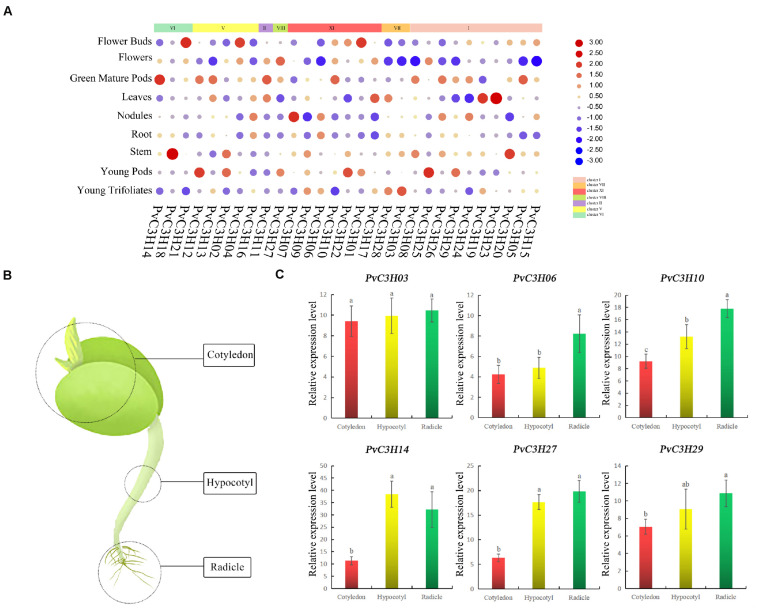
Expression profiles of *PvC3H*s. **(A)** Cluster analysis of expression patterns of *PvC3H*s during tissue development. *PvC3Hs* transcript in various tissues were retrieved from the Phytozome database. The data are presented as heat maps. The color scale indicates expression values; red denotes a high transcriptional level, while blue denotes a low transcriptional level. The circle size is proportional to the level of transcription; a larger circle indicates highly abundant transcripts. **(B)** Cartoon showing the tissues of the common bean during the sprout stage: cotyledon, hypocotyl, and radicle. *PvC3H*s having specific expression profiles were identified. **(C)** In total, nine *PvC3H* genes were selected randomly, and their expression levels in different tissues were investigated using the RT-qPCR method. Letters shown above the bars indicate significant differences (α = 0.05, LSD) among the treatments. The *x*-axis indicates sampling time, and the *y*-axis indicates relative gene expression.

The transcriptional patterns of *PvC3H*s in many tissues, including flower buds, flowers, green mature pods, leaves, nodules, roots, trifoliates, stems, pods, and seeds, were examined using high-throughput sequencing data from the Phytozome database. The expression levels of *PvC3H*s in various tissues were high, and the expression differences were relatively great (Figure 7A). To elucidate the functions of the *C3H*s in *P. vulgaris* at the sprout stage, six *PvC3H* genes were chosen randomly for analysis. RT-qPCR analyses of three tissues (cotyledon, hypocotyl, and radicle; Figure 7B) from R during the sprout stage was performed to assess the expression levels of these *PvC3H* genes on the fifth day. As shown in Figure 7C, the relative expression levels of some *PvC3H*s in the radicle and hypocotyl were relatively high, while the expression levels of other *PvC3H*s showed no differences among the three tissues. Therefore, the hypocotyl and radicle can be used as sprout tissues for *PvC3Hs* research.

### Expression Analysis of *PvC3Hs* in R and N Under Salt-Stress Conditions

**FIGURE 8:**
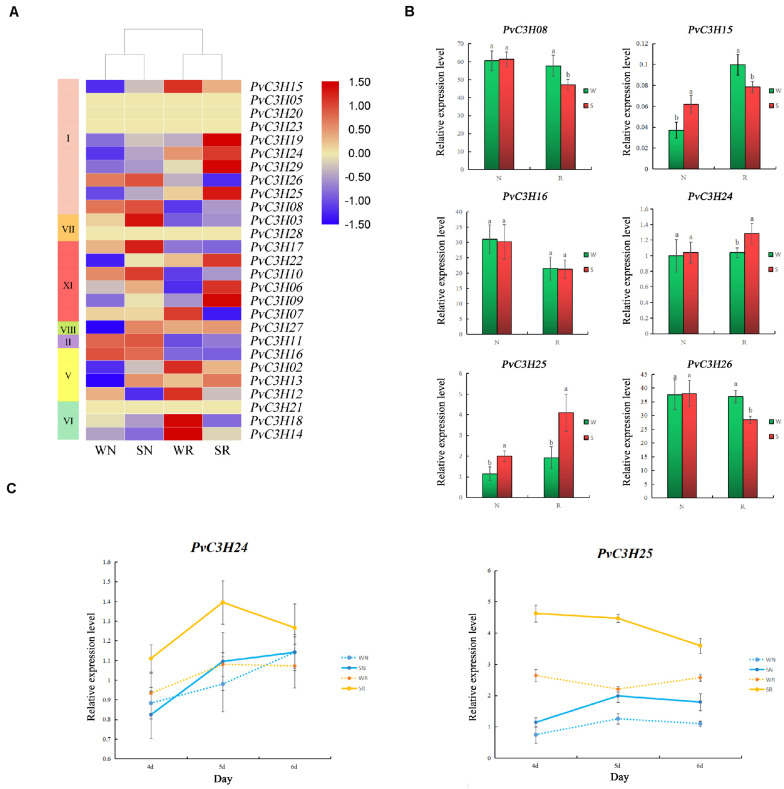
Transcriptome analysis of salt-tolerant (R) and salt-sensitive (N) common bean varieties under salt-stress conditions. The *x*-axis indicates sampling time, and the *y*-axis indicates relative gene expression. **(A)** A heat map with clustering was constructed using *PvC3H*s in the different varieties under salt-stress conditions. The color scale varies from blue to red, representing relatively low to high expression, respectively. **(B)** Six *PvC3H*s were selected and their expression assessed by RT-qPCR to determine the reliability of the transcriptome, the *y*-axis indicates relative gene expression. Lower case letter(s) above bars indicate significant differences (α = 0.05, LSD) between the treatments. **(C)** The expression changes of the two *PvC3H*s on the fourth, fifth, and sixth day. The yellow line represents R, and the blue line represents N; The *x*-axis indicates sampling time, and the *y*-axis indicates relative gene expression.

Two common bean varieties were tested to response to salt stress. R and N, were subjected to two treatments, W and S, and were analyzed after 5 days. The assembled gene dataset, deposited at the National Center for Biotechnology Information under the accession number PRJNA558376^15^, was used as a reference for further analyses (Supplementary Table 5). Using the constructed bean-free hypocotyl transcriptome, differences in *PvC3H* gene expression levels were identified (Figure 8A). Six *PvC3H*s were randomly selected for RT-qPCR analyses, and the results under W and S conditions were consistent with the transcriptome results (Figure 8B). Compared with N, the relative expression levels of the *PvC3H*s showed more differences between WR and SR. Two *PvC3H*s were selected randomly and RT-qPCR was performed using samples taken over 3 days. Although the change trend of the curve was different, the *PvC3H*s in R showed greater changes in expression under the W and S treatments compared with those in N (Figure 8C). Thus, under salt-stress conditions, the differential expression of *PvC3H*s may affect the salt tolerance of plants.

### GO Enrichment of *PvC3H* Genes’ Expression Analysis

**FIGURE 9:**
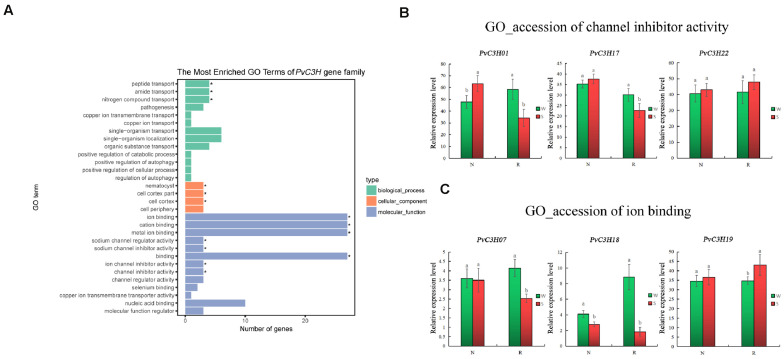
GO verified results of *PvC3H* enrichment. **(A)** Enrichment of GO terms in the transcriptome. The *P*-values for binding and channel activity are less than 0.05, reaching significance. **(B,C)** RT-qPCR analysis of *PvC3H* genes enriched in binding and channel activity. Letters above bars indicate significant differences (α = 0.05, LSD) between the treatments. The *x*-axis indicates sampling time, and the *y*-axis indicates relative gene expression.

The GO enrichment analysis of *PvC3H*s showed that they were enriched in many terms, such as ion binding, cation binding, metal ion binding, amide transport, sodium channel regulator, inhibitor activities, binding, and ion channel inhibitor activity (Figure 9A). Two GO categories were enriched after screened: ion binding and channel activity. Three genes enriched in the two terms were randomly selected for RT-qPCR verification. For the three *PvC3H*s enriched in channel activity (*PvC3H01*, *PvC3H17*, and *PvC3H22*; Figure 9B), and the three genes enriched in ion binding (*PvC3H07*, *PvC3H18*, and *PvC3H19*; Figure 9C), the differences in R between the W and S treatments were greater those in N. Thus, these genes may affect the expression of salt-tolerance traits during the sprout stage.

**FIGURE 10:**
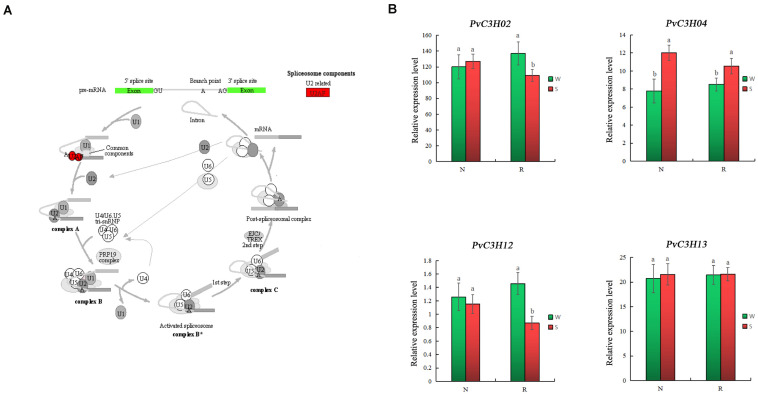
KEGG verified results of *PvC3H* enrichment. **(A)** Schematic diagram of the *PvC3H*s enriched in the “Spliceosome” (pvu03040) pathway. **(B)** RT-qPCR analysis *PvC3H* expression in the “Spliceosome” (pvu03040) pathway. Lower case letter(s) above bars indicate significant differences (α = 0.05, LSD) between the treatments. The *x*-axis indicates sampling time, and the *y*-axis indicates relative gene expression.

The KEGG analysis of *PvC3H*s showed that they were enriched in the “Spliceosome” (pvu03040) pathway in common bean, having a corrected *P*-value of 7.03e^–8^. The *PvC3H*s belonged to the U2-related module in the spliceosome (Figure 10). The RT-qPCR analysis of radicles from the two varieties showed that, compared with the S treatment, the changes in some genes’ expression levels (*PvC3H02*, *PvC3H04*, *PvC3H12*, and *PvC3H13*) in R were greater than in N, which indicated that the change in the abscisic acid content affected the expression levels of the *PvC3H* genes under salt-stress conditions, which may further affect the salt tolerance of the common bean during the sprout stage.

## Discussion

### Characterization of C3H Gene Family Members in Common Bean

Here, 29 *PvC3H*s were recognized from the genome of *P. vulgaris* L, suggesting that the *C3H* gene family has a wider distribution in common bean than in *A. thaliana* or rice. The *PvC3H* family was separated into seven subfamilies on the basis of the phylogenetic analysis. A similar method was used to identify subfamilies in maize (Peng et al., 2012), citrus (Liu et al., 2014), and *Brassica rapa* (Pi et al., 2018). Furthermore, the phylogenetic relationships of the *PvC3H*s were confirmed using their conserved motifs and gene structures. A gene structure analysis revealed that the *PvC3H*s contain from 0 to 11 introns. Additionally, every subfamily exhibited a similar exon–intron organization and contained *cis*-regulatory elements. These results may help reveal the functions of various gene families. According to the conserved motif analysis, the C3H domain (motif 1) was common to all the PvC3H proteins. Each subfamily harbored similar motifs, and the motif sequences in each subfamily were relatively similar, which indicated that *PvC3H* subfamily members may be conserved. Generally, the same subfamily shared an analogous exon–intron organization, as well as motif composition, and this has also been observed in rapeseed, poplar (Chai et al., 2012), rice (Wang et al., 2008), chickpea (Pradhan et al., 2017), Solanum lycopersicum (Xu, 2014), and Medicago truncatula (Zhang et al., 2013).

A comparative genomic analysis was performed to assess the structure of the genome at the syntenic block level, which reveals features that are conserved in multiple genomes (Ghiurcuta and Moret, 2014). This enables functional information to be transferred from a known taxon to a less-studied taxon. In this study, collinearity revealed that some *PvC3H* genes may have been generated through gene duplication. Moreover, segmental duplication events were vital in *PvC3H* evolution. The *Arabidopsis* homologous gene *HSFC1* responds to stress and also has a binding effect (Gaudet et al., 2011). *AtC3H14*, which contains a C3H zinc-finger protein, also binds to its target mRNAs during *A. thaliana* growth (Kim et al., 2014). The *PvC3H* homologous genes in *Arabidopsis* produce JA to induce the biosynthesis of the metabolite Nδ-acety lornithine, which enhances the defense functions (Adewale et al., 2011).

Therefore, the collinear *PvC3Hs* of common bean revealed in this study may also be involved in plant resistance. Thus comparative genomic analyses between common bean and other species form an important basis for future studies on the roles of *PvC3H* genes.

### *PvC3H* Expression During the Sprout Stage

Salt, which acts as important abiotic stress in agricultural production (Liang et al., 2018), is different from other biological stresses. Salt stress may exist from the plant’s germination stage, through its growth, and into maturity. The sprout stage is the initial period of crop growth, and stress during this period negatively affects the entire growth period. Additionally, seed germination is a critical stage for crop establishment (Hubbard et al., 2012), and it is the least tolerant to abiotic stress (Patade et al., 2011). Various abiotic stresses, especially salt stress, reduce the germination rate and delay the germination of high-quality but salt-sensitive seeds (Khan and Gul, 2006; Ansari and Sharif-Zadeh, 2012; Fazlali et al., 2013; Thiam et al., 2013). Therefore, it is very important to analyze the expression levels of *PvC3H*s during the sprout stage.

In this study, through the germination of R and N during W and S treatments, day five was selected as the sampling time. The tissue-specific expression results showed that the hypocotyl and radicle may be used as the sampling materials when studying *PvC3H*s, and hypocotyl growth has also been reported to be affected by salt stress (Rossi et al., 2016).

### Enrichment of *PvC3Hs*

Recent evidence showed that *C3H* genes play crucial roles in the adaptation to various abiotic stresses, especially salt stress, by plants, such as *A. thaliana* (Seok et al., 2018), rice (Jan et al., 2013), tobacco (Guo et al., 2009), and sweet potato (Zhang et al., 2019), but there is limited information on common bean. Transcriptome data from R and N indicated that the expression levels of *PvC3H* genes may cause salt tolerance in common bean sprouts. *PvC3H*s were enriched in the binding and channel activity GO terms and in the “Spliceosome” pathway. A RT-qPCR analysis of the *PvC3H*s enriched in GO and KEGG analyses was conducted. The differences in the expression levels of *PvC3H*s in R were more significant than those in N, suggesting that *PvC3H*s participate in the enrichment of the pathway and traits. The pathways for inhibitor activities, binding, and ion channel inhibitor, which were enriched in the GO analysis have been reported in previous studies on salt tolerance in plants, such as *A. thaliana* (Li et al., 2014; Kim et al., 2019; Wang et al., 2019), rice (Mansuri et al., 2019), cotton (Mu et al., 2019), poplar (Gaudet et al., 2011), and alfalfa (Lai et al., 2014). The spliceosome pathway has anti-stress effects in *A. thaliana* (Feng et al., 2015; Narasimha et al., 2018), *O. sativa* (Ashwini et al., 2018), and *Cannabis sativa* (Liu et al., 2016). Additionally, *C3H* has been reported to enhance stress resistance through spliceosome drivenRNA splicing (Bogamuwa and Jang, 2014). Thus, the enrichment results of these analyses provide new ideas and valuable information for studying the relationship between *PvC3Hs* and salt stress.

## Conclusion

Despite the importance of *C3H* genes in responses to salt stress, the precise roles of *C3H* gene family members in common bean have not been elucidated. Here, a reference genome of common been was used to comprehensively analyze 29 *PvC3H*s, including the identification of sequences, construction of phylogenetic trees, characterization of motif composition, and analyses of *cis*-regulatory elements, gene structure, collinearity, and expression levels in different tissues during the sprout stage. RNA-seq and RT-qPCR analyses confirmed that the expression levels of the *PvC3H*s underwent greater changes in R than in N. Through a GO analysis and KEGG enrichment, the *PvC3H*s were found to be enriched in ion binding, channel regulator activity, and the spliceosome pathway. These findings will aid further studies on the *C3H* and other gene families in common bean.

## Data Availability Statement

The datasets presented in this study can be found in online repositories. The names of the repository/repositories and accession number(s) can be found in the article/Supplementary Material.

## Author Contributions

QZ and J-DD conceived the study and designed and managed the experiments. W-JZ, WL, and ZY provided the plant lines. SZ, HZ, LZ, YW, and QZ performed the trials and collected the data. ZY and QZ completed the statistical analysis of the phenotypic data and wrote the manuscript. All authors contributed to writing the manuscript.

## Conflict of Interest

The authors declare that the research was conducted in the absence of any commercial or financial relationships that could be construed as a potential conflict of interest.

## Footnotes

1 http://hmmer.janelia.org/

2 http://pfam.sanger.ac.uk/

3 http://plants.ensembl.org/index

4 http://www.ebi.ac.uk/interpro/

5 http://smart.embl-heidelberg.de/

6 http://prosite.expasy.org/

7 https://wolfpsort.hgc.jp/

8 http://www.p3db.org/

9 http://prosite.expasy.org/

10 http://gsds.cbi.pku.edu.cn/

11 http://meme.nbcr.net/meme/

12 http://circos.ca/

13 http://www.genome.jp/keg

14 https://phytozome.jgi.doe.gov/pz/portal.html

15 http://www.ncbi.nlm.nih.gov/bioproject/
