# Supplementary material for: Genome- and Transcriptome-Wide Identification of C3Hs in Common Bean (Phaseolus vulgaris L.) and Structural and Expression-Based Analyses of Their Functions During the Sprout Stage Under Salt-Stress Conditions
Source: Front Genet. 2020 Sep 15;11:564607. doi: 10.3389/fgene.2020.564607 (PMC7522512; doi:10.3389/fgene.2020.564607)
Supplement: Supplementary file 1 [file Table_1.doc]

**Supplementary Table 1. Quality analysis of the transcriptome data.**

| Sample name | Raw Reads | Clean Reads | Q20（%） | Q30（%） | Top Mapped（%） | Multiple Mapped（%） |
| --- | --- | --- | --- | --- | --- | --- |
| WN1 | 55633866 | 53026610 | 96.84 | 92.16 | 83.57 | 0.86 |
| WN2 | 50577884 | 48331946 | 96.69 | 91.84 | 84.8 | 0.86 |
| WN3 | 52446224 | 50061634 | 96.68 | 91.86 | 84.53 | 0.95 |
| SN1 | 41534300 | 40199494 | 94.93 | 88.25 | 78.62 | 0.81 |
| SN2 | 50772136 | 49248286 | 95.10 | 88.47 | 79.82 | 0.98 |
| SN3 | 48326882 | 46935654 | 95.13 | 88.53 | 80.32 | 0.87 |
| WR1 | 46820132 | 45146702 | 94.64 | 87.77 | 72.62 | 0.90 |
| WR2 | 43365056 | 41854676 | 94.77 | 87.98 | 72.36 | 1.03 |
| WR3 | 46518476 | 44905982 | 94.63 | 87.75 | 72.55 | 0.85 |
| SR1 | 46162584 | 44307832 | 94.59 | 87.82 | 72.06 | 0.77 |
| SR2 | 46908800 | 45348350 | 94.83 | 88.04 | 73.32 | 0.75 |
| SR3 | 45229736 | 43738928 | 95.02 | 88.49 | 73.9 | 0.71 |
