# Supplementary material for: Genome- and Transcriptome-Wide Identification of C3Hs in Common Bean (Phaseolus vulgaris L.) and Structural and Expression-Based Analyses of Their Functions During the Sprout Stage Under Salt-Stress Conditions
Source: Front Genet. 2020 Sep 15;11:564607. doi: 10.3389/fgene.2020.564607 (PMC7522512; doi:10.3389/fgene.2020.564607)
Supplement: Supplementary file 2 [file Table_2.doc]

| Primer | Sequence (5 'to 3') | Primer | Sequence (5 'to 3') |
| --- | --- | --- | --- |
| *Pvactin11-qF* | TGCATACGTTGGTGATGAGG | *PvC3H01-qF* | TCCTGGTGGCTATAATGCTGTT |
| *Pvactin11-qR* | AGCCTTGGGGTTAAGAGGAG | *PvC3H01-qR* | GATGCTTCTACGCTGATCTTGG |
| *PvC3H02-qF* | GCCTAGAAGCCGTAGTCCTC | *PvC3H03-qF* | TTCTTCGCCTTCTGCTCACT |
| *PvC3H02-qR* | GCACCACTCCGTTCTTGATAC | *PvC3H03-qR* | CTCATTCACCTGCTGCTCATC |
| *PvC3H04-qF* | CGAGGACTTCTACGAGGACAT | *PvC3H06-qF* | TGGAGTGTCTGATGCTGTAGG |
| *PvC3H04-qR* | GCTCTGGTTGATGTGGTAGTAG | *PvC3H06-qR* | CGGTGATAAGGTGGTAGTTGTG |
| *PvC3H07-qF* | TTCTCTGACCACATTCCATCCT | *PvC3H08-qF* | ATGATGGAGTGGAGACGAAGG |
| *PvC3H07-qR* | GCAATTCTCACCGTTCCTACAA | *PvC3H08-qR* | GTGCTGGTGCTGTTGTTGAA |
| *PvC3H10-qF* | AGTGCGTAGGTGTCATCAATC | *PvC3H12-qF* | ACCATTCTGCTCGCCAACA |
| *PvC3H10-qR* | GTGTGCTCTGTTAGTGTCTGTA | *PvC3H12-qR* | GGACTCCTGCTTCTGCTTCT |
| *PvC3H13-qF* | TCTGCGACAACCTTGCTGAT | *PvC3H14-qF* | AAGGCTGAAGTTGGACAAGTTG |
| *PvC3H13-qR* | TCCTCACTGCCATCTCTAACTG | *PvC3H14-qR* | TCCTCATCCTCCTCACAGTCA |
| *PvC3H15-qF* | GGACTGGCTTCTGTGGCTTT | *PvC3H16-qF* | GCAAGAGCAAGAGCGAGAG |
| *PvC3H15-qR* | AGTTTAGCGTCACAGGGGCA | *PvC3H16-qR* | CCGATTCCGATTCCGATACG |
| *PvC3H17-qF* | ACCTCTGGCTGTCCATTCG | *PvC3H18-qF* | CTTCCGCAGCCTCCACATT |
| *PvC3H17-qR* | CGTCCACCTATCCTGCTGTT | *PvC3H18-qR* | CCTCCACGCACATATACCTTCT |
| *PvC3H19-qF* | GCAAGGATGATTGGCGAGTT | *PvC3H22-qF* | GCTCTGCTCCATCTGCTGTTA |
| *PvC3H19-qR* | CGGCTGAGGATGGTGGAAT | *PvC3H22-qR* | GTCTCGTGCTCTCGGATTGA |
| *PvC3H24-qF* | TTCTGGTGACAACATTGAAGGT | *PvC3H25-qF* | GCTTCTTCGCTCACACCAA |
| *PvC3H24-qR* | GGTGATGGTAGCGACAGTTAC | *PvC3H25-qR* | CTCTTCTTCCTCACACGCATT |
| *PvC3H26-qF* | AGGTTGATGGTGGTGGTGAT | *PvC3H27-qR* | GGTGGCAGTTGGAGAAGGA |
| *PvC3H26-qR* | ATTGAGGATGCTGATGAAGACA | *PvC3H27-qR* | ACAATGCTCGCAGAAGTAGTG |
| *PvC3H29-qF* | ACTTGCCGCTACAATCATCCT |  |  |
| *PvC3H29-qR* | TGCCATCTCCACAACTTCTCC |  |  |

**Supplementary Table 2. RT-qPCR primer design.**
