# Supplementary material for: Genome- and Transcriptome-Wide Identification of C3Hs in Common Bean (Phaseolus vulgaris L.) and Structural and Expression-Based Analyses of Their Functions During the Sprout Stage Under Salt-Stress Conditions
Source: Front Genet. 2020 Sep 15;11:564607. doi: 10.3389/fgene.2020.564607 (PMC7522512; doi:10.3389/fgene.2020.564607)
Supplement: Supplementary file 3 [file Table_3.doc]

**Supplementary Table 3. *Cis*-regulatory element analysis and functional predictions of *PvC3H* gene family members.**

| Element | Core sequence | Classification | Function |
| --- | --- | --- | --- |
| GARE-motif | TCTGTTG | Hormone related elements. | Gibberellin-responsive element. |
| MBS | CAACTG | Resistance-related elements. | MYB binding site involved in drought-inducibility. |
| TCA-element | CCATCTTTTT | Hormone related elements. | *Cis*-acting element involved in salicylic acid responsiveness. |
| TATC-box | TATCCCA | Hormone related elements. | *Cis*-acting element involved in gibberellin-responsiveness. |
| ARE | AAACCA | Resistance-related elements. | *Cis*-acting regulatory element essential for the anaerobic induction. |
| ABRE | ACGTG | Hormone related elements. | *Cis*-acting element involved in the Abscisic acid responsiveness. |
| AuxRR-core | GGTCCAT | Hormone related elements. | *Cis*-acting regulatory element involved in auxin responsiveness. |
| TGA-element | AACGAC | Hormone related elements. | Auxin-responsive element. |
| LTR | CCGAAA | Resistance-related elements. | *Cis*-acting element involved in Low-temperature responsiveness. |
| P-box | CCTTTTG | Hormone related elements. | Gibberellin-responsive element. |
| CAT-box | GCCACT | Elements related to sprout stage. | *Cis*-acting regulatory element related to meristem expression. |
| RY-element | CATGCATG | Elements related to sprout stage. | *Cis*-acting regulatory element involved in seed-specific regulation. |
| GC-motif | CCCCCG | Resistance-related elements. | Enhancer-like element involved in anoxic specific inducibility. |
