# Supplementary material for: Genome- and Transcriptome-Wide Identification of C3Hs in Common Bean (Phaseolus vulgaris L.) and Structural and Expression-Based Analyses of Their Functions During the Sprout Stage Under Salt-Stress Conditions
Source: Front Genet. 2020 Sep 15;11:564607. doi: 10.3389/fgene.2020.564607 (PMC7522512; doi:10.3389/fgene.2020.564607)
Supplement: Supplementary file 4 [file Table_4.doc]

Supplementary Table 4. Ka and Ks calculations for *PvC3H* tandem repeat gene pairs.

| Genes pair | Ka | Ks | Ka/Ks |
| --- | --- | --- | --- |
| PvC3H01&PvC3H22 | 0.479732 | 1.65204 | 0.290387 |
| PvC3H13&PvC3H12 | 0.0160208 | 0.0711886 | 0.225047 |
| PvC3H23&PvC3H19 | 0.94696 | 1.18332 | 0.800254 |
