# Supplementary material for: Genome- and Transcriptome-Wide Identification of C3Hs in Common Bean (Phaseolus vulgaris L.) and Structural and Expression-Based Analyses of Their Functions During the Sprout Stage Under Salt-Stress Conditions
Source: Front Genet. 2020 Sep 15;11:564607. doi: 10.3389/fgene.2020.564607 (PMC7522512; doi:10.3389/fgene.2020.564607)
Supplement: Supplementary file 5 [file Table_5.doc]

**Supplementary Table 5. The accession numbers for transcriptome data.**

| Bioproject accession | Biosample accession | Sample name | Library ID | Title |
| --- | --- | --- | --- | --- |
| PRJNA558376 | SAMN12405822 | SAMN12405822 | library_01 | WN |
| PRJNA558376 | SAMN12405823 | SAMN12405823 | library_02 | SN |
| PRJNA558376 | SAMN12405826 | SAMN12405826 | library_05 | WR |
| PRJNA558376 | SAMN12405827 | SAMN12405827 | library_06 | SR |
